# Therapeutic Notes

**Published:** 1896-12-26

**Authors:** 


### THERAPEUTIC NOTES.

### ENTEROL.

Enterol[1] is a new antiseptic for internal use, and possessing particular advantages in the treatment of septic conditions of the urinary tract, especially in pyelitis, cystitis, gonorrhœal and surgical kidney. It is a combination of the various cresols which are formed under normal conditions by the metabolism of food stuffs in the lower part of the bowel, and which act as natural antiseptics and prevent further decomposition. These cresols, whether artificially ingested or naturally formed, are absorbed chiefly by the lacteals, and finally eliminated in the urine as ethereal sulphates. During their passage along the urinary tract in the form of ethereal sulphates, they exercise a salutary antiseptic action on the mucous membrane or epithelial lining of the bladder, renal tubes and pelvis of the kidney, &c. In septic conditions of these parts its action is said to be more efficacious than salicylic or benzoic acids. Enterol is a thin liquid of a light brown colour, with characteristic odour and soluble in 100 parts of water. As an antiseptic it is said to be six times as active as carbolic acid. For practical purposes a solution of 1 in 500 is found sufficiently strong, and of this one drachm is the usual dose. It can be taken regularly for a considerable time without producing any disagreeable symptoms. It can be taken in the form of pill or capsule, and may be conveniently combined with an equal quantity of olive oil. The urine of persons under a course of treatment with this drug does not undergo the usual ammoniacal fermentation, and may stand for many days without showing signs of decomposition. Doubtless enterol has many other useful applications, either as a local or general antiseptic; but for septic condition of the urinary tract it is stated to be so far superior to other bodies of a similar nature, that in this department we may expect to find it of the greatest service.

[1] Med. Modern, Oct 31, 1896.

### NOTES ON THE SERUM TREATMENT OF DIPHTHERIA.

In the *Therapeutic Monatsheft* for February, 1896, there is an exhaustive summary and bibliography of the literature of the above subject, dealing with a period of nine months from April till the previous December. With regard to the mortality statistics it appears that nearly all authorities are agreed that when the serum treatment is applied within the first twenty-four hours of the onset of symptoms, a cure of the condition is practically ensured. Behring's (*Therapeutic Monatsheft*, Feb., 1896) statistics for Berlin show that when employed in the first twenty-eight hours the mortality for all cases was as low as 7·3 per cent. Although these statistics may be taken as substantially correct, Gottstein (*Therapeutic Monatsheft*, Feb,, 1896) questions their accuracy, and Healy (*Medical Record*, Nov. 17th, 1896) attributes the general lowering of the mortality to a natural fall in the curve, the amplitude of which shows differences from year to year. The time of disappearance of the membrane from the commencement of treatment appears to differ widely according to different observers, its range oscillating between twelve hours and fourteen days. With regard to the temperature, opinion here again differs widely: while some notice a lowering, others record a rise. Variot believes that the effect on the temperature differs according to the age of the patient. In children there is often a rise, accompanied by other symptoms. Soltmann and Kurt Muller (*Berlin Klin. Wochensel*, No. 137, 1896) have noticed an almost universal initial rise of temperature. There appears to be a somewhat universal agreement with regard to the increased frequency of post-diphtheritic paralysis after the serum treatment, and Monte gives a full account of this complication (*Wein. Med. Woch.*, No. 4, 1895). There are other complications which occur with considerable frequency during the course of the treatment. An erythematous rash is perhaps the most common. It may assume a form like measles, scarlatina, or urticaria. Œdema of the limbs is another complication occasionally met with, a condition which is described by Konigshöfe. Urinary troubles are also by no means uncommon, for suppression of urine, oliguria, and albuminuria have all been noticed. In the same number the prophylactic treatment by Behring's serum is also fully dealt with, and, speaking generally, most of the authorities there quoted are agreed that the practice is by no means justifiable.

